# Can Caregiver Reports Reflect Dental Treatment Needs of Patients with Intellectual and Developmental Disabilities?

**DOI:** 10.3290/j.ohpd.b972947

**Published:** 2021-02-23

**Authors:** Juhea Chang

**Affiliations:** a Clinical Professor, National Dental Care Center for Persons with Special Needs, Seoul National University Dental Hospital, Seoul, Korea.

**Keywords:** caregiver, caries, DMFT, intellectual disability, oral health, periodontal index

## Abstract

**Purpose:**

This study aimed to obtain the oral health-related factors of patients with intellectual and developmental disabilities (IDD) from family caregivers and to relate caregiver-perceived risk factors to dental treatment needs of patients.

**Materials and Methods:**

A total of 120 dyads of patients (mean [SD] age = 29.1 [8.4] years) and their family caregivers (mean [SD] age = 56.5 [9.5] years) were included. Data were obtained from self-administered questionnaires by caregivers and oral examinations by a dentist. Oral health conditions of patients were analysed in different age groups using paired t-tests. Caregiver-perceived oral health conditions of patients and dentist-assessed caries and periodontal disease were compared using Pearson’s chi-squared and Fisher’s exact tests. Relationships between patient factors and treatment needs were analysed using multiple logistic regression.

**Results:**

Tooth pain, chewing difficulty, and reasons for the last dental visit were associated with high numbers of decayed teeth (DT) (p < 0.05). Overall oral health condition of patients rated by caregivers was related to high DT and the Community Periodontal Index (CPI) score (p < 0.05). Well-maintained dental care of caregivers was associated with lower numbers of DT and less urgent treatment needs of patients (p < 0.05).

**Conclusion:**

There were caregiver-perceived factors indicating dental treatment needs of patients with IDD. Proxy reports by caregivers can be used as risk predictors for ongoing dental problems of patients with communication limitations.

People with intellectual and developmental disabilities (IDD) are prone to oral health problems, resulting in a higher prevalence of periodontal disease and caries development compared to their non-disabled counterparts.^6^ Caregivers are often frustrated by barriers imposed on dental treatment of patients with IDD despite their high treatment needs.^3^ Among the many obstacles, communication limitations in particular are known to be the main challenge for patients with IDD who require health care provision.^14^ These patients are in a similar situation to young children who are uncooperative due to avoidance behaviour.^15^ Adolescent and adult patients have permanent dentition that can present with more complications and extensive problems than children, resulting in more serious consequences of unmet treatment needs. Moreover, engagement by dental practitioners with patients with IDD is difficult to initiate because preoperative screenings such as medical and dental histories, determining problem severity, and performing oral examinations and other evaluations, are restricted. At this point, caregiver reports can be useful to provide valid information on the oral health status of patients and to assist in professional assessments.

Regarding with proxy reports of oral health conditions, most studies have focused on young children with their parents. A previous study by this author included the patients with intellectual disabilities cared for by professional caregivers in institutional facilities.^4^ It demonstrated that oral hygiene and periodontal disease were similarly rated both by caregivers and dentists, but assessments for decayed and missing teeth were influenced by patient and caregiver circumstances. This study provided more detailed questionnaires and conducted oral examinations in a clinical setting. In addition, the study population included family caregivers that were more knowledgeable about their patients and mainly assisted in their daily activities. The aims of the study were firstly to evaluate how family caregivers reported the oral health conditions of patients with IDD, and secondly to determine how patient and caregiver factors were related to dentist-assessed disease status and treatment urgency. The hypothesis was that caregiver-perceived factors would be indicative of dental treatment needs of patients with IDD.

## Materials and Methods

### Study Design

This study included a total of 120 dyads of patients (mean ± SD age/range = 29.1 ± 8.4)/13–59 years) with intellectual disabilities and their main caregivers (mean ± SD age/range = 56.5 ± 9.5/22–83 years). The patients received dental treatment under general anaesthesia (GA) at the National Dental Care Center for Persons with Disabilities, Seoul National University Dental Hospital from June 2018 through February 2020. The inclusion criteria were as follows: family caregivers of (1) patients older than 12 years and (2) patients deemed ineligible for conventional dental treatment due to their intellectual and cognitive limitations. Exclusion criteria were as follows: family caregivers (1) accompanying the patients but who were not the primary caregivers, and (2) declining to participate in the study or not comprehending the questionnaires. The study design was thoroughly explained to the participating caregivers and approved with their written consents. Data obtained from (1) oral examinations conducted by a dentist and (2) survey questionnaires answered by a caregiver constituted a dyad. The Seoul National University Dental Hospital Institutional Review Board approved the study (CRI18006).

### Questionnaires

The self-administered questionnaires included items on sociodemographic characteristics, oral health conditions, and dental behaviours of both patients and caregivers. Four subsets of independent variables were included: (1) patient demographic factors, (2) patient dental factors, (3) caregiver demographic factors, and (4) caregiver dental factors. Demographic factors for patients were sex, age, disabilities (onset, type, and severity), medications, GA history, meal types, assistance of daily living, and caregiver types. Dental factors for patients were as follows: pain in teeth, untreated cavities, missing teeth, gum bleeding with and without toothbrushing, toothbrushing pattern, amount of saliva, chewing and swallowing difficulty, experience of scaling, dental flossing, cooperation with dental treatment, intervals and reasons for dental visits, last cavity treatment, and overall impression of oral health status. Demographic factors for caregivers were sex, age, marital status, education, patient relationships, occupation, and economic status. Dental factors for caregivers were the same as those for patients except for cooperation toward dental treatment.

### Oral Examination

The index of DMFT (decayed, missing, and filled teeth) was determined based on WHO criteria.^20^ Decayed teeth (DT) were recorded as present when a lesion in a pit or fissure or on a smooth tooth surface has an unmistakable cavity, undermined enamel, or a detectably softened floor or wall. Teeth with carious lesions in contact with previous restorations (secondary caries) were also recorded as DT. Teeth lost due to periodontal issues and injury were not counted as missing teeth (MT). The decision was based on clinical documentation including caregiver interviews and clinical and radiographic examination under GA. Periodontal status was recorded using the Simplified Oral Hygiene Index (OHI-S) and Community Periodontal Index (CPI). The OHI-S was the sum of the Debris Index and Calculus Index on six representative tooth surfaces, with each index scored from 0 (no debris and calculus) to 3 (debris and calculus covering more than 2/3 of tooth surfaces).^11^ Six surfaces were selected from four posterior and two anterior teeth for examination. In posterior teeth, the buccal surfaces of the maxillary first molars and the lingual surfaces of the manidbular first molars were chosen. In anterior teeth, the labial surfaces of the maxillary right and the mandibular left central incisors were chosen. In the absence of the above-mentioned teeth, the adjacent molars and anterior teeth on the opposite side were selected instead. The CPI evaluated gingival bleeding (CPI = 1), calculus deposition (CPI = 2), and deep probing pocket depths (4–5mm, CPI = 3 and ≥6 mm, CPI = 4).^2^ Dental erosion and trauma status were recorded by severity and number of involved teeth. Finally, treatment urgency was rated as follows: 1 = preventive or routine treatment needed, 2 = prompt treatment including scaling needed, and 3 = immediate treatment needed due to pain or infection of dental and/or oral origin.^20^

### Statistical Analysis

Caries experience and periodontal index were compared using t-tests between the two age groups (< 30 and ≥ 30 years). Pearson’s chi-squared test and Fisher’s exact test were used to compare proportions among the patient and caregiver characteristics for subcategories of disease status. Multiple logistic analysis was used to determine relationships between patient factors and treatment needs. For this analysis, samples that contained any missing or unknown values (unmarked or answered with ‘I don’t know’) were excluded. Also, all independent variables were included in regression analysis, except those that were predominantly categorised into a single type (patients’ GA history, main caregiver type, and caregivers’ marital status) or too diverse to be categorised (caregivers’ occupation). Data were analysed using SAS version 23.0 (SAS Institute; Cary, NC, USA) with alpha = 0.05.

## Results

Table 1 compares caries and periodontal conditions in two age groups of patients (< 30 years vs ≥ 30 years). The mean (SD) numbers of DT and DMFT of the patients were 3.83 (4.06) and 10.77 (7.01), respectively. DT, MT, FT, and DMFT did not differ statistically significantly between the two groups. Patients with CPI scores of 1, 2, or ≥ 3 comprised 7.5%, 76.7%, and 15.8% of total patients, respectively. The mean (SD) CPI score was 2.35 (0.65) in the group ≥ 30 years and 1.92 (0.59) in the group < 30 years, which was a statistically significant difference (p < 0.05).

**Table 1:** Oral health conditions of patients included in the study

Age	Mean (SD) numbers of teeth with caries experience				Mean (SD) scores of the periodontal index				Dental trauma^§^ (%)
	DT	MT	FT	DMFT	Debris index	Calculus index	Oral hygiene index	CPI	
< 30 years	3.52 (3.87)	1.02 (2.15)	5.36 (4.88)	10.05 (7.36)	0.29 (0.10)	0.25 (0.14)	0.29 (0.10)	1.92 (0.59)	16.7
≥ 30 years	4.20 (4.28)	1.39 (2.00)	6.06 (4.45)	11.65 (6.53)	0.28 (0.10)	0.29 (0.11)	0.28 (0.10)	2.35 (0.65)	11.7
Total	3.83 (4.06)	1.18 (2.08)	5.68 (4.68)	10.77 (7.01)	0.28 (0.10)	0.27 (0.13)	0.28 (0.10)	2.12 (0.65)	28.3
p-values	0.36	0.33	0.42	0.21	0.52	0.05	0.52	< 0.01	0.69*

^§^Prevalence of patients having experienced traumatic dental injury. *p-value from chi-squared tests.

The caregiver response rates were dissimilar in each part of the questionnaires (Tables 2 to 4). The response rates were 100% for patient and caregiver demographic factors, 56.7% to 96.7% for patient dental factors, and 78.3% to 100% for caregiver dental factors. Table 2 shows the distribution of caries incidence related to caregiver-reported patient characteristics. Tooth pain, chewing difficulty, poorer oral health conditions, and recent history of dental visit other than check-ups were related to higher numbers of DT (p < 0.05). Table 3 shows the distribution of periodontal conditions. Gums that bled without brushing were associated with higher scores of OHI-S (p < 0.05). Poorer-rated oral health conditions were associated with higher CPI scores (p < 0.05). Table 4 shows urgent treatment needs confirmed by dentist assessments. Among the cases, treatment urgency levels 1, 2, and 3 comprised 5.0%, 53.3%, and 41.7%, respectively. Factors contributing to treatment urgency level 3 as opposed to levels 1 or 2 were sex, tooth pain, bleeding gums, chewing difficulty, poorer-rated oral health condition, and recent dental visits due to pain or discomfort (p < 0.05).

**Table 2:** Distribution of caries incidence related to caregiver-reported dental characteristics of patients

Patient characteristics		No. of cases	Decayed teeth (DT), % of cases				p-values
			0	1~2	3~5	>5	
Tooth pain	No	35	31.4	45.7	17.1	5.7	<0.01
	Yes	52	13.5	23.1	26.9	36.5	
Bleeding gums on brushing	Never or sometimes	52	21.2	38.5	17.3	23.1	0.29
	Often or always	68	13 (19.1)	17 (25.0)	20 (29.4)	18 (26.5)	
Bleeding gums without brushing	Never	71	13 (18.3)	28 (39.4)	16 (22.5)	14 (19.7)	0.09
	Sometimes, often, or always	49	11 (22.4)	9 (18.4)	13 (26.5)	16 (32.7)	
Chewing difficulty	No	41	12 (29.3)	13 (31.7)	11 (26.8)	5 (12.2)	0.03
	Yes	66	9 (13.6)	21 (31.8)	13 (19.7)	23 (34.8)	
Amount of saliva	Insufficient	25	6 (24.0)	6 (24.0)	5 (20.0)	8 (32.0)	0.49
	Normal or sufficient	38	5 (13.2)	14 (36.8)	10 (26.3)	9 (23.7)	
Overall oral health condition	Very poor	30	4 (13.3)	5 (16.7)	4 (13.3)	17(56.7)	<0.01
	Poor	52	9 (17.3)	19 (36.5)	16 (30.8)	8 (15.4)	
	Average or good	37	11 (29.7)	13 (35.1)	8 (21.6)	5 (13.5)	
Reasons for the last dental visit	Check-up, cleaning, or scaling	54	12 (22.2)	17 (31.5)	19 (35.2)	6 (11.1)	0.03
	Problems other than pain	41	6 (14.6)	12 (29.3)	7 (17.1)	16 (39.0)	
	Pain or discomfort	25	6 (24.0)	8 (32.0)	3 (12.0)	8 (32.0)	

**Table 3:** Distribution of periodontal health conditions related to caregiver-reported dental characteristics of patients

Patient characteristics		No. of cases	Simplified Oral Hygiene Index (OHI-S), % of cases				Community Periodontal Index (CPI), % of cases		
			< 2	2–4	≥ 4	p-values	1–2	3–4	p-values
Tooth pain	No	35	17.1	60.0	22.9	0.76	85.7	14.3	0.71
	Yes	52	11.5	63.5	25.0		82.7	17.3	
Bleeding gums on brushing	Never or sometimes	52	19.2	55.8	25.0	0.23	84.6	15.4	0.91
	Often or always	68	8.8	66.2	25.0		83.8	16.2	
Bleeding gums without brushing	Never	71	19.7	57.7	22.5	0.05	84.5	15.5	0.90
	Sometimes, often, or always	49	4.1	67.3	28.6		83.7	16.3	
Chewing difficulty	No	41	14.6	61.0	24.4	0.93	85.4	14.6	0.78
	Yes	66	12.1	63.6	24.2		83.3	16.7	
Amount of saliva	Insufficient	25	16.0	68.0	16.0	0.48	88.0	12.0	1.00
	Normal or sufficient	38	15.8	55.3	28.9		86.8	13.2	
Overall oral health condition	Very poor	30	10.0	63.3	26.7	0.73	80.0	20.0	0.03
	Poor	52	11.5	59.6	28.8		76.9	23.1	
	Average or good	37	18.9	62.2	18.9		97.3	2.7	
Reasons for the last dental visit	Check-up, cleaning, or scaling	54	13.0	53.7	33.3	0.34	85.2	14.8	0.43
	Problems other than pain	41	14.6	70.7	14.6		87.8	12.2	
	Pain or discomfort	25	12.0	64.0	24.0		76.0	24.0	

**Table 4:** Patient characteristics according to treatment urgency by dentist assessment

Patient characteristics		No. of cases	Treatment urgency^§^, % of cases		p-values
			1 or 2	3	
Sex	Male	76	65.8	34.2	0.03
	Female	44	45.5	54.5	
Age (years)	<30	66	60.6	39.4	0.58
	≥30	54	55.6	44.4	
Tooth pain	No	35	80.0	20.0	<0.01
	Yes	52	40.4	59.6	
Bleeding gums on brushing	Never or sometimes	52	59.6	40.4	0.01
	Often or always	68	57.4	42.6	
Bleeding gums without brushing	Never	71	67.6	32.4	0.01
	Sometimes, often, or always	49	44.9	55.1	
Chewing difficulty	No	41	68.3	31.7	0.09
	Yes	66	51.5	48.5	
Amount of saliva	Insufficient	25	72.0	28.0	0.12
	Normal or sufficient	38	52.6	47.4	
Overall oral health condition	Very poor	30	23.3	76.7	<0.01
	Poor	52	63.5	36.5	
	Average or good	37	81.1	18.9	
Reasons for the last dental visit	Check-up, cleaning, or scaling	54	75.9	24.1	<0.01
	Problems other than pain	41	46.3	53.7	
	Pain or discomfort	25	40.0	60.0	

^§^Intervention urgency by the WHO criteria. 1 = preventive or routine treatment needed; 2 = prompt treatment (including scaling) needed; 3 = immediate (urgent) treatment needed (pain/infection of dental and/or oral origin).

In simple logistic regression analysis, several factors among patient and caregiver characteristics were detected with relation to DT, CPI, and treatment urgency (p < 0.05, Table 5). Multiple logistic regression analysis revealed significant predictors of DT and treatment urgency (Table 6). Tooth pain was related to higher numbers of DT (p < 0.05). Bleeding gums without brushing and poorer-rated oral health conditions were related to higher treatment urgency (p < 0.05).

**Table 5:** Simple logistic regression analysis of patient and caregiver characteristics related to treatment needs

Treatment needs	Characteristics		Odds ratio	95% CI	p-values
DT (number of decayed teeth)	**Patient-related**				
	Type of care	Home and daycare (Ref)			
		Home	3.37	1.02–11.13	0.05
	Type of meals	Normal consistency (Ref)			
		Soft or liquid	2.62	1.10–6.22	0.03
	Tooth pain	No (Ref)			
		Yes	5.86	2.22–15.46	<0.01
	Overall oral health condition	Average or good (Ref)			
		Poor	1.58	0.66–3.77	0.30
		Very poor	4.31	1.53–12.09	0.01
	**Caregiver-related**				
	Economic status	Good (Ref)			
		Average	3.29	1.16–9.35	0.03
		Poor	4.50	1.39–14.61	0.01
	Last scaling	< 6 months (Ref)			
		6-12 months	1.18	0.48–2.90	0.72
		≥ 12 months	2.45	1.03–5.81	0.04
	Untreated cavity	No (Ref)			
		Yes	2.41	1.04–5.58	0.04
CPI 0 = no periodontal ds. 1 = bleeding 2 = calculus 3 = pocket 4–5mm 4 = pocket ≥ 6mm	**Patient-related**				
	Age	< 30 (Ref)			
		≥ 30	4.27	1.43–12.78	0.01
	Overall oral health condition	Average or good (Ref)			
		Poor	10.80	1.34–87.24	0.03
		Very poor	9.00	1.02–79.55	0.05
Treatment urgency 1 = preventive 2 = prompt 3 = urgent	**Patient-related**				
	Sex	Male (Ref)			
		Female	2.31	1.08–4.93	0.03
	Disability type	Intellectual disability (Ref)			
		Developmental disability	0.41	0.17–0.99	0.05
		Others	0.68	0.27–1.70	0.41
	Caring type	Home and daycare (Ref)			
		Home only	6.00	1.30–27.74	0.02
	Reasons for the last dental visit	Check-up, cleaning, or scaling (Ref)			
		Problems other than pain	3.65	1.52–8.76	0.00
		Pain or discomfort	4.73	1.72–13.05	0.00
	Tooth pain	No (Ref)			
		Yes	5.91	2.18–15.99	0.00
	Bleeding gums without brushing	No (Ref)			
		Yes	2.56	1.21–5.43	0.01
	Overall oral health condition	Average or good (Ref)			
		Poor	2.47	0.91–6.69	0.08
		Very poor	14.08	4.33–45.83	<0.01
	**Caregiver-related**				
	Last scaling	< 6 months (Ref)			
		6-12 months	1.26	0.50–3.20	0.62
		> 12 months	2.47	1.00–5.87	0.04

**Table 6:** Multiple logistic regression analysis of caregiver-perceived patient characteristics related to DT, CPI, and treatment urgency

Treatment needs	Patient characteristics		Odds ratio	95% CI	p-values
DT (number of decayed teeth)	Sex	Male (Ref)			
		Female	0.94	0.30–2.92	0.91
	Age (years)	<30 (Ref)			
		≥30	0.99	0.35–2.82	0.99
	Tooth pain	No (Ref)			
		Yes	4.38	1.30–14.71	0.02
	Bleeding gums with brushing	No (Ref)			
		Yes	1.46	0.51–4.22	0.48
	Bleeding gums without brushing	No (Ref)			
		Yes	1.33	0.42–4.16	0.63
	Chewing difficulty	No (Ref)			
		Yes	1.34	0.40–4.46	0.64
	Overall oral health condition	Average or good (Ref)			
		Poor	2.60	0.73–9.34	0.14
		Very poor	3.70	0.81–16.83	0.09
	Reasons for the last dental visit	Check-up, cleaning, or scaling (Ref)			
		Problems other than pain	0.45	0.12–1.69	0.24
		Pain or discomfort	0.36	0.10–1.33	0.13
CPI 0 = no periodontal ds. 1 = bleeding 2 = calculus 3 = pocket 4–5mm 4 = pocket ≥ 6mm	Sex	Male (Ref)			
		Female	1.63	0.40–6.66	0.50
	Age (years)	<30 (Ref)			
		≥30	3.62	0.80–16.52	0.10
	Tooth pain	No (Ref)			
		Yes	0.58	0.13–2.66	0.48
	Bleeding gums with brushing	No (Ref)			
		Yes	1.50	0.37–6.13	0.57
	Bleeding gums without brushing	No (Ref)			
		Yes	1.86	0.45–7.78	0.39
	Chewing difficulty	No (Ref)			
		Yes	1.23	0.25–5.97	0.80
	Overall oral health condition	Average or good (Ref)			
		Poor	7.67	0.80–73.25	0.08
		Very poor	4.54	0.35–58.15	0.25
	Reasons for the last dental visit	Check-up, cleaning, or scaling (Ref)			
		Problems other than pain	0.60	0.10–3.46	0.57
		Pain or discomfort	2.00	0.39–10.15	0.41
Treatment urgency 1 = preventive 2 = prompt 3 = urgent	Sex	Male (Ref)			
		Female	1.57	0.46–5.36	0.47
	Age (years)	<30 (Ref)			
		≥30	0.72	0.22–2.38	0.59
	Tooth pain	No (Ref)			
		Yes	3.29	0.84–12.84	0.09
	Bleeding gums with brushing	No (Ref)			
		Yes	1.32	0.42–4.18	0.64
	Bleeding gums without brushing	No (Ref)			
		Yes	3.94	1.21–12.86	0.02
	Chewing difficulty	No (Ref)			
		Yes	0.72	0.19–2.76	0.63
	Overall oral health condition	Average or good (Ref)			
		Poor	1.90	0.46–7.95	0.38
		Very poor	5.58	1.01–29.23	0.04
	Reasons for the last dental visit	Check-up, cleaning, or scaling (Ref)			
		Problems other than pain	1.42	0.35–5.70	0.62
		Pain or discomfort	2.92	0.74–11.52	0.13

## Discussion

This study investigated how family caregivers perceived the oral health status of patients with IDD, and how caregiver perception reflected the treatment needs of the patients. Several symptomatic characteristics of patients reported by caregivers coincided with advanced caries and periodontal disease, resulting in urgent treatment needs.

Previous studies on the proxy reports of oral health conditions have mostly focused on the parents of young children with early childhood caries.^5,9,10,18^ The literature is very scarce on adolescent and adult populations suffering from IDD with the associated communication limitations. In this age group, permanent dentition is established, and dental problems become complicated when periodic check-ups and interventions are deficient.^17^ Global epidemiological studies indicate that caries prevalence peaks at 25 years of age, largely because young adults neglect oral health maintenance after leaving school. This situation becomes more serious for the adult population with IDD, because they tend to resist oral-hygiene measures such as toothbrushing, and parental guidance decreases as the patients age. Therefore, oral hygiene maintenance and acceptance of professional dental care become more difficult for those with IDD. Another challenge of this study group is the severity of communication limitations. Intellectual disabilities (ID) are largely heterogeneous in nature, ranging from mild to profound.^19^ Most oral health studies have included populations with a mild degree of communication deficiency and adequate cooperation for examinations – one example being groups with Down Syndrome. These mildly ID groups can be investigated on a larger scale and more similar to epidemiological research on the general population. In this study, patients were suffering from a severe degree of ID and behavioural issues, and neither home nor professional dental care were easily achievable. Their treatment needs involved more serious concerns, because access to dental professionals was limited, and ongoing problems were aggravated with time. Since patients’ dependence on others for daily living activity is considerable, the caregivers’ responsibilities for oral health care are critical.^16^ It was first hypothesised that caregivers could perceive dental problems suffered by their patients, even though such patients were not able to clearly express their feelings. As a result, caregiver-perceived symptoms would be indicative of existing diseases and treatment needs.

In this study, main family caregivers were able to detect patients’ symptoms associated with presence of caries and periodontal diseases. Conventionally, higher incidence of toothaches among young children is regarded as an indicator of insufficient dental care.^1,13^ This study clearly supported that tooth pain was the most pronounced symptom associated with multiple areas of untreated decay and urgent treatment needs. In this study, many carious teeth required endodontic treatment due to the extent of the carious lesions. These advanced cases of caries contributed to the presence of painful teeth and higher treatment needs (treatment urgency level 3). Bleeding without brushing was shown to be a more significant sign than that with brushing, representing poor oral hygiene status (high OHI-S). As an established measurement, bleeding upon probing is an early and sensitive sign of gingival inflammation.^12^ Particularly for the IDD group with poor oral hygiene, bleeding gums can be an easy predictor of periodontal problems detected by a third person. It is readily recognisable by caregivers, because patients are often assisted during feeding, washing, and toothbrushing. Despite insufficient toothbrushing, most patients did not show severe periodontal breakdown. The mean (SD) CPI score was 2.12 (0.65), and only 15.8% of cases exhibited development of periodontal disease (CPI ≥ 3). However, higher CPI scores were more pronounced in the older group (≥ 30 years). A previous study on age-related oral health status of adolescents and adults with ID demonstrated that poor plaque and gingival indices were more evident in older (≥ 20 years) than younger (< 20 years) groups.^17^ Therefore, even the initial stage of periodontal problems (gingival bleeding and calculus deposition) in young adults with IDD should be carefully monitored in risk assessment. Additionally, caries experience did not differ between age groups, revealing similarly high unmet treatment needs in general. Altogether, the hypothesis of this study was accepted.

Another intriguing question was whether caregiver factors would influence the patients’ oral health conditions. In the author’s previous study, institutional caregivers of adults with ID exhibited different oral health perceptions according to their own dental care habits and working conditions.^4^ It was determined that family caregivers can be more attentive to patients, and their awareness of oral health conditions may be less influenced by circumstances. In addition, the dental behaviours of family-member caregivers would have a greater effect on the oral health profiles of their patients than other (professional) caregivers. In this study, family caregivers rated the overall oral health conditions of their patients relative to the severity of problems, such as the presence of multiple untreated carious lesions, advanced periodontal disease, and overall treatment urgency. Regarding the dental attitudes and behaviours of parents and children, it was common to find that the parents were more attentive to their own oral health care, and that their children exhibited better profiles of oral health status.^7,8^ In this study, the patients were middle-aged, and the influence of parental guidance was less than in the case of young children. Still, the patients showed lower treatment urgency, since the caregivers received their last scaling more recently (Table 5). Caregivers’ well-established oral health habits motivated them to seek dental care for their patients, despite patients’ insufficient cooperation. Other favourable indicators of oral health maintenance of caregivers, such as better economic status and absence of untreated caries, were also related to the lower treatment needs of patients.

Considering the severity of intellectual and cognitive deficiency of patients, seeking health care services is mainly dependent on the caregivers’ decision. Nevertheless, caregivers have limited knowledge of their patients’ problems, and it makes them unable to properly ask for intervention. This study attempted to incorporate as many variables as possible that could be influenced by the main caregivers who assist in the daily activities of their patients. However, some questions seemed difficult for caregivers to answer. For example, 43.3% responded as ‘don’t know’ to the question such as ‘do you think your patient has enough saliva in his/her mouth?’ In the statistical analysis, all missing or unanswered responses were excluded, resulting in heterogeneous response rates depending on the nature of questions. In future studies, the sample sizes must be increased and more relevant factors should be revealed in association with progressing disease status. When a structured questionnaire containing items related to clinically relevant variables is provided, caregivers can become better aware of the seriousness of their patients’ oral health conditions and effectively deliver the information to dental practitioners. In addition, caregivers’ awareness will motivate them to strive for more optimal oral health outcomes in their patients. Eventually, lifetime cost and risk of dental treatment will be reduced for this susceptible special-needs population.

## Conclusion

Family-member caregivers were able to detect the dental problems of patients with severe IDD. Tooth pain, gums bleeding without brushing, and poor oral health condition were representative indicators of treatment urgency of patients. Caregiver reports of oral health status can be useful for predicting dental treatment needs and motivating oral health maintenance of patients with intellectual and developmental limitations.
